# Information and Discriminability as Measures of Reliability of Sensory Coding

**DOI:** 10.1371/journal.pone.0001328

**Published:** 2007-12-19

**Authors:** Jan Grewe, Matti Weckström, Martin Egelhaaf, Anne-Kathrin Warzecha

**Affiliations:** 1 Department of Neurobiology, Bielefeld University, Bielefeld, Germany; 2 Psychological Institute II, Westfalian Wilhelms-University Münster, Münster, Germany; 3 Department of Biophysics and Biocenter Oulu, University of Oulu, Oulu, Finland; Centre de Recherches su la Cognition Animale - Centre National de la Recherche Scientifique and Université Paul Sabatier, France

## Abstract

Response variability is a fundamental issue in neural coding because it limits all information processing. The reliability of neuronal coding is quantified by various approaches in different studies. In most cases it is largely unclear to what extent the conclusions depend on the applied reliability measure, making a comparison across studies almost impossible. We demonstrate that different reliability measures can lead to very different conclusions even if applied to the same set of data: in particular, we applied information theoretical measures (Shannon information capacity and Kullback-Leibler divergence) as well as a discrimination measure derived from signal-detection theory to the responses of blowfly photoreceptors which represent a well established model system for sensory information processing. We stimulated the photoreceptors with white noise modulated light intensity fluctuations of different contrasts. Surprisingly, the signal-detection approach leads to a safe discrimination of the photoreceptor response even when the response signal-to-noise ratio (SNR) is well below unity whereas Shannon information capacity and also Kullback-Leibler divergence indicate a very low performance. Applying different measures, can, therefore, lead to very different interpretations concerning the system's coding performance. As a consequence of the lower sensitivity compared to the signal-detection approach, the information theoretical measures overestimate internal noise sources and underestimate the importance of photon shot noise. We stress that none of the used measures and, most likely no other measure alone, allows for an unbiased estimation of a neuron's coding properties. Therefore the applied measure needs to be selected with respect to the scientific question and the analyzed neuron's functional context.

## Introduction

Some of the most fundamental questions in neuroscience address the stimulus features encoded by a sensory system, the amount of information that can be transmitted given the neuronal response variability, the timescale on which relevant information is encoded, and the nature of the neural code. Widely applied measures have been derived from information theory [1] and signal-detection theory [2]. Information theory has been applied to quantify the amount of information conveyed by neuronal responses [3]–[6] or to characterize the reliability of synaptic transmission [7], [8]. Measures of the discriminability of neuronal responses have been applied, for instance, to estimate the relevant timescale of neuronal coding [9], [10] or to quantify the response reliability [11], [12]. Both types of reliability measures, i.e. the information theoretical and the signal-detection ones, shed light on the accuracy with which a sensory system encodes stimuli. However, it is still not clear how these measures are related and whether their application leads to equivalent conclusions.

**Figure 1:**
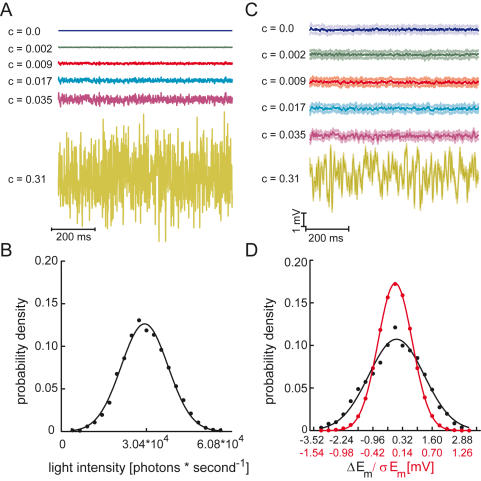
Stimulus and response properties. A Stimulus traces of different contrasts. The contrast, c, is defined as the ratio of standard deviation of the light intensity modulation and the mean light intensity (3.04*10^4^ effective photons per second and receptor). The light intensity values were drawn from a normal distribution and the sequences were lowpass filtered with a 2nd order Butterworth filter with 256 Hz cut-off. B Probability density of the 0.31 contrast stimulus with a Gaussian fit. C Typical responses of a photoreceptor to the contrast modulation illustrated in A [averages over 25 trials (dark colors)±the standard deviation (light colors)]. D Probability density functions of photoreceptor response (black dots and black labels on x-axis) and the response noise (red dots and red labels on x-axis) fitted with Gaussian functions.

In the present account we compared estimates of system performance obtained from information theory (Shannon information capacity, Kullback-Leibler divergence) and a discrimination method derived from signal-detection theory. All measures are applied to the same set of neuronal responses. Our study was done on an intensively investigated model system for sensory information processing, the photoreceptors in the blowfly (*Calliphora vicina*) retina [7], [13], [14]. Single photoreceptors were stimulated with Gaussian distributed random light intensity fluctuations of different contrasts (figure 1A, B) superimposed on a background luminance. The photoreceptor responses were analyzed with the different measures and the corresponding results were compared.

We find that responses to very weak stimuli can be safely discriminated with the signal-detection analysis, whereas the information theoretical approaches indicate that much stronger stimuli are needed to markedly increase the Shannon information capacity or Kullback-Leibler divergence of the responses. Thus, the use of different measures leads to very different conclusions about the ability of photoreceptors to encode luminance changes. This becomes particularly obvious when assessing the impact of photon shot noise on the reliability of the photoreceptor responses. Photon shot noise reflects the physical limitation on accuracy of a visual system resulting from the random emission of photons from a light source. Its importance has been investigated in various accounts based on analyses of the signal-to-noise ratio and was concluded to be a major, but not the only, source of photoreceptor response variability [7], [XPATH ERROR: unknown variable "rids-text".]. Our discrimination analysis supports these results but suggests a much stronger impact of photon noise than estimated before or suggested by the presented information theoretical approaches.

## Results

### Information theoretical analyses

**Figure 2:**
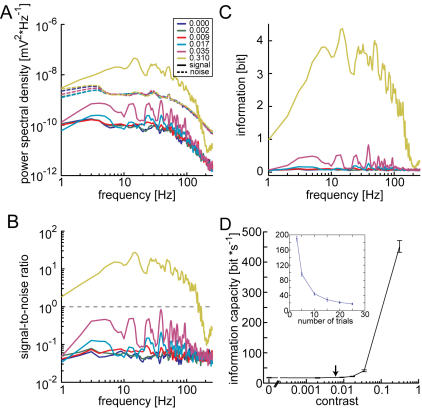
Information theoretical analysis. A S(f), the power spectral density of the signal (mean response, solid lines), and N(f), the power spectral density of noise (dashed lines), normalized to the signal or noise mean square amplitude. The inset assigns different colors to the different stimulus contrasts. B Signal-to-noise ratios at the six different contrasts used (same color code as in A). Dashed line indicates SNR of 1. C Shannon information as function of frequency at different contrasts (same color code as in A). Information estimated from signal-to-noise ratio as: log_2_[1+S(f)/N(f)]. All spectra were smoothed using a 4 point running average. D Shannon information capacity as a function of contrast; average ±95% confidence interval (N = 12). At the 0.31 contrast 14 additional cells were analyzed. Abscissa is interrupted to display zero contrast on the logarithmic scale. Arrow marks the contrast induced by photon shot noise at the mean light intensity. Inset shows the dependence of the information capacity on the amount of data analyzed. The information capacities shown were calculated at zero contrast by using different numbers of trials to estimate the SNR.

Single photoreceptors were stimulated with Gaussian distributed random light intensity fluctuations of different contrasts (‘c’, defined as the ratio of standard deviation and mean luminance; figure 1A, B) superimposed on a background luminance. The applied contrasts ranged from zero (no modulation) to the average contrast of natural scenes (c_natural_ = 0.31) [16]. With increasing contrast the response amplitude increases, but the standard deviation representing response variability stays about the same (figure 1C). Accordingly, the noise power spectral densities (N(f), dashed lines figure 2A) are largely independent of the respective stimulus contrast indicating the additive nature of the noise. Both the mean response as well as the membrane voltage noise are normally distributed and can be fitted well with a Gaussian function (figure 1D). Since S(f) (solid lines in figure 2A) increases with increasing contrast while the different N(f) are largely independent of the contrast, the SNR (figure 2B) increases with increasing stimulus contrast in accordance with previous investigations [15]. For all tested contrasts but the largest (0.31) the SNR is well below unity for the entire frequency range, indicating that the noise component dominates the individual photoreceptor responses. The Gaussian distribution of signal and noise, the additive nature of the noise, and the almost linear light intensity coding in blowfly photoreceptors [17] allowed us to calculate the Shannon information capacity [18] as a measure of coding performance (see Methods). Parallel to the SNR the amount of transmitted information and the bandwidth in which information is transmitted increases with contrast (figure 2D). The total information capacity (equation 3) increases with increasing contrast, initially very slowly and steeply only beyond the contrast of 0.035 (figure 2D). At zero contrast the measurement of an information capacity larger than zero is an artifact and the consequence of limited data. In case of an unlimited amount of data information capacity at zero contrast is zero. The inset in figure 2D illustrates this dependence on the amount of data and shows the alleged information capacity estimated at zero contrast as a function of the number of trials evaluated. As can be seen, the information capacity is high for a small number of trials and declines with an increasing amount of data. The 25 trials (rightmost data point in the inset) used for our analyses appear appropriate for a good SNR estimation. The analysis of the data on the basis of the information capacity indicates that information about stimuli with a contrast below 0.035 is hardly transmitted at all, because signals appear to be buried in noise.

An information theoretical approach to the question of whether two signals are distinguishable or not is the Kullback-Leibler divergence which is related to the mutual information [3], [19]. We apply the Kullback-Leibler divergence to assess the discriminability between the responses used above (which we will call in the following the *reference stimuli*) and the responses to random luminance sequences with statistically the same contrast and cut-off frequency but with different time course at each presentation (subsequently called *test stimuli*, see methods). The Kullback-Leibler divergence compares two distributions and is zero only for exactly matching distributions and differs from zero for diverging distributions. Hence, a larger divergence indicates better discriminability of the responses.

**Figure 3:**
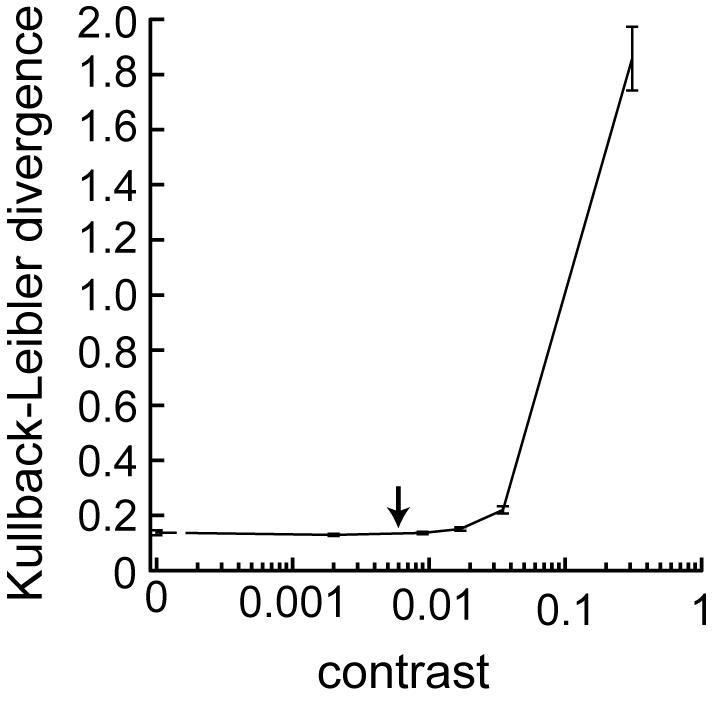
Kullback-Leibler divergence as a function of the contrast. Average Kullback-Leibler divergence ±95% confidence interval at the different contrast (N = 12). For each recorded cell the Kullback-Leibler divergence was estimated at each instance of time and was subsequently averaged across time giving a single divergence value for each cell at each contrast level (see methods). The plotted values are averages of these divergences across the 12 cells.

Figure 3 shows the Kullback-Leibler divergence as a function of stimulus contrast. Like the Shannon information capacity it increases only very slightly at first and its largest increment is beyond a contrast of 0.035. Thus, with this information theoretical measure of discriminability similar conclusions about the system's response reliability could be drawn as with the information capacity.

### Signal-detection analysis

**Figure 4:**
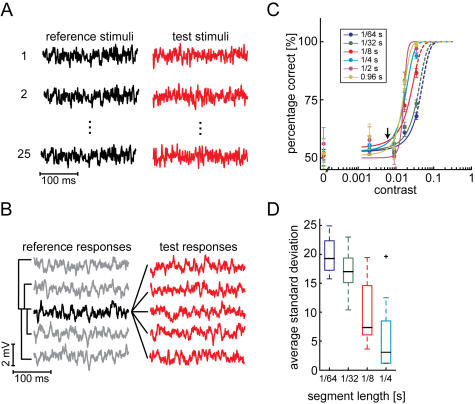
Signal-detection approach. A Experimental design for the discrimination task at one contrast level. Left hand column: the reference stimulus is repeated 25 times; right hand column: different test stimuli, all statistically equivalent to the reference stimulus. Reference and test stimuli of all five contrasts were presented in a pseudorandom order. B At each contrast level the two distances 〈*D_r_*〉 and 〈*D_t_*〉 were estimated according to equation 5 for each reference response (e.g. the highlighted one in the left box). C Response discriminability as a function of contrast. Dots mark the average discrimination performance (N = 12) ±SEM. Discrimination performances were calculated using data segments of different lengths (see legend). Data were fitted with a sigmoid function ranging from 50 to 100% (equation 6). The arrow marks the contrast which results from photon shot noise at the background light intensity. D Uncertainty of discriminability estimation for different segment lengths. For each cell, the discrimination performance was estimated for all possible data segments. The standard deviation of these discrimination performances was estimated. The box plots describe the distribution of these standard deviations in the cell population. Boxes indicate median (black line) and the upper, respectively lower quartile the whiskers represent the rest of the data. The plus-sign denotes an outlier. The two largest segment lengths used in C subdivided response traces only into one or two segments, thus no S.D. could be calculated.

In a discrimination approach derived from signal-detection theory we ask how well the fly's photoreceptor responses to a certain sequence of light intensities could be discriminated from those to different sequences. This approach is similar to the one used to evaluate the impact of photon noise on the reliability of the spike responses of the motion sensitive H1-cell downstream in the fly's visual system [11]. Here we compare the responses to 25 repetitions of a *reference stimulus* to those evoked by the test stimuli, i.e. 25 different white noise sequences with the same statistical properties like cutoff frequency and contrast (figure 4A). For this analysis we used the same data as for the application of the Kullback-Leibler divergence while the information capacity was estimated on the basis of the *reference responses* only. The discrimination measure calculates the response discriminability and its rationale will be briefly sketched in the following (for details see methods).

In the case of a noise free encoding system even slightly different stimuli lead to different responses. In other words: the *reference responses* should be identical and each *test response* should be clearly different from the *reference responses* as well as from the other *test responses*. Analogously, the difference (estimated as the distance, equation 5) between a certain *reference response* and the other *reference responses* should be zero while it should be larger than zero when comparing to the *test responses*. In a real system with noise, these assumptions do not hold and the distances of a certain *reference response* to the other *reference responses* and also to the different *test responses* are larger than zero. Since the *reference responses* were evoked by repetitions of the same stimulus the distances between the *reference responses* should still be smaller than those to the test responses once the stimulus induced response is strong enough. The discrimination performance was defined as the proportion of *reference responses* for which this assumption holds. One major advantage of this discrimination method is its independence of assumptions about the statistics of the underlying data. Thus, also responses evoked by natural contrast modulations which are clearly different from white noise [14] can be analyzed in this way; an approximation of the Shannon information capacity for such stimuli would require much more data since the simplifying assumptions of Gaussian distribution and coding linearity do not hold for natural stimuli.

As expected from the increasing response amplitude and power (figure 1C and 2A, respectively), the discriminability increases with increasing contrast. Indeed the discrimination performance increases with contrast and has its strongest increment at contrasts exceeding only 0.009 and a performance of 75% correct decisions is achieved at a contrast of only about 0.014 (figure 4C yellow curve; estimated from the sigmoid fit, equation 6). An almost perfect discrimination performance could be observed already at a contrast as low as 0.035. Therefore, discrimination performance was not determined for larger contrasts. Anyway, this signal-detection analysis appears more sensitive to detect stimulus induced changes in the responses than the applied information theoretical measures.

### Dependence of coding performance on available time

In normal behavioral situations, animals have only limited time to distinguish meaningful stimuli on the basis of neuronal responses contaminated with noise. Therefore, the dependence of the performance on the duration of the evaluated data segments has to be considered. The different measures of coding performance depend differently on the length of the data segments. In case of the information capacity and the Kullback-Leibler divergence it is not the performance per se that degrades with decreasing segment length but mainly the accuracy of the estimation. The signal-detection approach also suffers from a reduced segment length as shown in figure 4C, however in a different way: with short segments, the discrimination performance is reduced and this reduction is accompanied by increased uncertainty of discrimination performance (figure 4D). This less reliable estimation of the response distances with short segments is partly responsible for the reduction in discrimination performance. Due to the asymmetry of the discrimination measure the discriminability may well drop below 50% but cannot exceed 100% and therefore introduces a bias to lower performances. In any case, our discrimination approach shows insignificant differences in discrimination performance once the segment length is sufficient to reliably estimate the distance between responses traces (i.e. at segment lengths exceeding 125 ms) indicating the validity of the estimation of discrimination performance with the available amount of data.

### Impact of photon noise

Photon shot noise is an unavoidable noise source in visual systems and the question about its importance for visual performance has been discussed for a long time [11], [20]–[22]. Owing to the random emission of photons the light intensity of a light source varies about the mean light intensity. The amplitude of these photon noise induced fluctuations can be quantified by their standard deviation and, if related to the mean, their contrast.
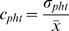
(1)with σ*_pht_* being the standard deviation and 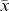 the mean number of effective photons per second. Accordingly the dependencies of the different measures of coding performance on the contrast of the added brightness fluctuations can be related to this contrast induced by photon shot noise (c_pht_). The stimulus used in our experiments had a mean brightness of about 30,000 effective photons per receptor and second. This estimate is based on counting distinct depolarizations of the photoreceptor membrane potential evoked by single photons at very low stimulus intensities and linear extrapolation to higher intensities. Photon emission is a random process characterized as a Poisson process with a variance equal to the mean. Hence, σ*_pht_* can be directly estimated from the mean brightness:
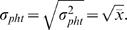
(2)


Thus, by inserting equation 2 into equation 1 the contrast induced by photon shot noise can be, roughly, approximated at the mean brightness of 30,000 effective photons per second to be: cpht = 0.006 (arrows in figure 2D, 3, 4C).

Contrasts just exceeding c_pht_ can already be discriminated on the basis of the photoreceptor responses by employing our signal-detection approach. This finding suggests that photon noise is a major source of variability limiting the precision of photoreceptor responses (figure 4C). The Shannon information capacity and the Kullback-Leibler divergence, on the other hand, have their strongest increments at much larger contrasts (figure 2D). The information theoretical approaches thus suggest a much smaller impact of photon noise under the light levels of the experiment and underestimate its importance dramatically.

## Discussion

In the present study we have applied different measures of neuronal response reliability to evaluate the coding performance of blowfly photoreceptors. Our results show that the application of information theoretical measures like the Shannon information capacity and the Kullback-Leibler divergence on the one hand, and the signal-detection theory based discriminability measure on the other, leads to very different conclusions about the photoreceptor response reliability. All measures indicate increasing performance with increasing stimulus contrast but with different sensitivity. Already small contrasts lead to major increases in response discriminability in the signal-detection approach but increase the Shannon information capacity and the Kullback-Leibler divergence only little. This sensitivity difference results in dramatically different conclusions about the importance of photon shot noise for photoreceptor coding performance.

Comparing results gained from the Shannon-information capacity and our signal-detection approach might look like the comparison of apples and oranges. In particular, the information capacity is usually applied to evaluate a system's encoding capabilities while signal-detection measures are commonly used to assess the decoder's side of neuronal information processing. Increased response reliability, however, increases the SNR, leads to an increased information capacity and, in parallel, increases response discriminability in the signal-detection task. We believe that these measures, although addressing seemingly different aspects of neuronal information processing, can be employed to tackle the problem of coding performance since they depend similarly on response quality.

It would be a different matter if our discrimination approach would look for certain features in the responses and thereby implement a detection task. In our discrimination approach, the “decoder” is the distance estimation (equation 5), a standard root-mean-square distance, which does not assume a certain response distribution or the presence of features that are to be recognized. One implication of equation 5 is that it gives larger deviations a stronger weight than smaller ones. However, removing this imbalance by estimating the average absolute of point-to-point differences does not change the results (not shown). Hence, even though other distance measures could have been applied, it is implausible that the exact realization of the distance estimation strongly affects the estimated response discriminability. Moreover, even if there were a clearly superior distance measure to the one we have chosen, we already find with our distance measure a much higher sensitivity than with the information theoretical approaches tested.

The high discrimination performance, exceeding 75% correct, in the signal-detection approach indicates that the responses to stimuli of a contrast as low as 0.017 can be faithfully discriminated (figure 4D). This finding leads us to the somewhat astonishing conclusion that the temporal structure of the responses can safely be discriminated already at contrasts where the SNR is much smaller than unity. In accordance with the very poor SNR at this contrast the Shannon information capacity suggests that the responses contain hardly any information (figure 2D). Also the Kullback-Leibler divergence as an information theoretical discrimination measure indicates a very poor discriminability at the contrast of 0.017 (figure 3). Both information theoretical measures depend similarly on the contrast and show major increments at much larger contrasts than the signal-detection analysis (compare figs. 2D, 3 and 4D, respectively). We decided to base our interpretations on the increments of measured performance rather than on significances since significances depend on the sample size. With the signal-detection approach we obtain a value of discriminability of the individual trials.

In the signal-detection analysis the performance is related to the highest possible performance, i.e. 100% correct. For the information capacity and also the Kullback-Leibler divergence we do not know what the right reference might be. We chose to refer the measured values to the performance measured at the average contrast of natural scenes. Compared to the performance at this contrast the gain of information capacity at a contrast of 0.017, for example appears negligible (information capacities of 4.7±1.79 and 432.2±57,79 bit/s above the zero contrast measures for contrasts of 0.017 and 0.31, respectively). Selecting the natural contrast as reference might appear arbitrary, but this contrast is widely used to characterize photoreceptor responses and the information capacity obtained for c = 0.31 is still not the highest possible. Higher capacities have been found at larger contrasts and higher light levels [7]. Since using a higher contrast as reference would lead to an even smaller relative increment of the information capacity at low contrast, the impact of photon noise on the coding performance of photoreceptors as assessed on the basis of the Shannon information and thus the discrepancy to the conclusions based on the signal-detection approach are not overestimated.

In both information theoretical approaches we observe an information capacity, or, respectively, divergence that is larger than zero also at zero contrast. This is due to the unreliable estimations of either the SNR [14] in case of the Shannon information capacity or the response distributions of *reference* and *test responses* in case of the Kullback-Leibler divergence. Controls in which we manipulate the SNR reliability by varying the amount of analyzed data show that this zero-contrast performance can be treated as a baseline (not shown) justifying to analyze the performances at larger-than-zero contrasts relative to the zero-contrast performance.

The contrasts levels at which the applied measures show increasing performance suggest a different importance of photon shot noise for the photoreceptor response reliability. All measures indicate that photon noise plays a role, but from the information capacity and the Kullback-Leibler divergence one would have underestimated its importance and accordingly overestimated the amount of noise originating within the photoreceptor itself. The role of photon shot noise depends on the light level. Our analysis indicates that it is very prominent under our experimental conditions (about 30,000 effective photons per second). At dimmer light levels photon noise can be expected to be even more prominent since its relative contribution increases with decreasing number of photons. At higher light levels when the signal-to-noise ratio of the visual input signal is higher, photon noise was shown to be negligible [15].

Here we found considerable differences between the different measures of coding performance on the very first stage of visual information processing, i.e. the photoreceptor level. Similar differences have been found in the electrosensory organ of the weakly electric fish [23]. There, signal-detection and information theoretical approaches gave seemingly contradicting results after a sensory signal underwent dendritic filtering. The choice of the appropriate measure, out of the variety of possible measures [19] depends very much on the scientific question. If, for instance, the question were how many different stimulus levels could be coded given the observed neuronal noise, the information capacity may be well suited. On the other hand, if we assume any decoding mechanism that takes temporal characteristics into account, information capacity would not be the measure of choice, since it considers only the frequency content of the responses assuming their independence and not their phase relations. Instead, the signal-detection approach appears appropriate to quantify the system's performance in representing the time course of a stimulus. It should be noted that contrasts exceeding 0.035 lead to saturated discrimination performance whereas information capacity and Kullback-Leibler divergence appear to increase significantly only above this saturating contrast level. Hence for stimuli of larger contrasts the signal-detection analysis gives no additional clues about the system's capabilities to discriminate the time course of the responses.

Although all applied methods are artificial measures of system performance, the distance estimation (equation 5) could well be neuronally implemented. All essential parts of the distance measure, i.e. the subtraction and the correlation of two inputs signals are implemented for example in circuits involved in motion detection [24]. Additionally, with a data segment of 125 ms, approximating a behaviorally relevant time for a fly to evaluate optic flow [25], i.e. the interval between subsequent saccadic turns characterizing fly orientation behavior, two signals can be discriminated faithfully at a contrast of only 0.025 (75% correct discrimination performance). In this range the Shannon information capacity and the Kullback-Leibler divergence indicate hardly any increase.

The results presented here demonstrate that different measures applied to the same data can yield different answers to the same question. This further demonstrates the difficulty in combining knowledge obtained with different measures. Neither of the used methods, however, gives a complete description of the systems performance.

## Methods

### Electrophysiology

Experiments were carried out on female blowflies (*Calliphora vicina*). The retina was accessed through a small hole cut into the fly's eye on the equatorial line close to the lateral rim which was sealed with silicon grease to prevent drying up. Sharp electrodes (Clark GC-150) were pulled on a Brown-Flaming P-97 Puller (Sutter Instruments) to have resistances of 80 to 90 MΩ when filled with 2 M KCl. Recordings were done in bridge mode using a SEC-10L amplifier (npi electronics, Tamm, Germany). We accepted recordings with a dark adapted membrane potential lower than −50 mV, a saturating light response of at least 50 mV and an input resistance of at least 25 MΩ. Responses were sampled at 4096 Hz (DAQBoard 2000, IOtech, Cleveland, OH) and stored on hard disk for offline analysis.

### Light stimulation

Photoreceptors were stimulated using LEDs (3 mm diameter, 525 nm light emission, type: WU-14-730GC, Vossloh-Schwabe Optoelectronic GmbH, Germany, covering approximately 1.15° of visual space). Since the data were collected as part of a study on motion vision two LEDs were used separated by 3°. One of them was positioned in the optical axis of the recorded cell. The off-axis LED had only little impact on the responses of the recorded cell (control experiments with only a single LED revealed no different results). LEDs were driven by a voltage-to-current converter controlled by the analogue outputs of the data acquisition board and they were used in the linear range of the current-light characteristic. Light intensities were calibrated by counting single photon responses at very low light intensities obtained with neutral density filters (Lee Filters, UK) in front of the LED. The average light intensity was estimated to 3.04*10^4^ effective photons per receptor and second. Receptors were stimulated with band limited (256 Hz cut-off) white noise light intensity modulations of different contrasts (figure 1).

### Data analysis

Data analysis was done in MATLAB (The Mathworks, Natick, MA). For the analysis the responses were converted to deflections relative to the membrane potential at constant background illumination. To estimate the Shannon information capacity the responses were segregated into the stimulus-induced response component (referred to as signal) and the stimulus-independent response component (referred to as noise). The across trial average of the 25 stimulus presentations was regarded as the signal while the difference between each individual response trace and the across trial average was assumed to represent the noise. The inset in figure 2D shows that 25 trials deliver an appropriate approximation of signal and noise. Signal and noise power spectral densities [S(f) and N(f)] for all but the largest contrast were calculated from 4000 data point segments (963,379 ms) zero padded to 4096 data points (1s) windowed with a 4096 point Hanning window. Power spectra were normalized to the mean square amplitude of the data. At the largest contrast three such segments with 50% overlap were used, again a 4096 point Hanning window was applied. A similar method was used with the smaller data segments.

### Estimation of the Shannon information capacity

Since signal and noise response components are Gaussian distributed and independent and the response amplitude linearly depends on the light level the Shannon information capacity [1], R, could be easily calculated from the ratio of the average signal power spectral density and noise power spectral density [S(f) and N(f), respectively]:
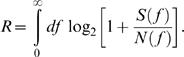
(3)


### Kullback-Leibler divergence

For the Kullback-Leibler divergence [3], [19] and the signal-detection analysis (see below) two types of responses were recorded: 1^st^, the *reference responses* which are 25 repetitions of the same stimulus at each contrast level. Those responses were used to calculate the Shannon information capacity (above). 2^nd^ the *test responses* which were 25 responses to 25 different random light sequences with statistically the same contrast and cutoff frequency as the reference stimuli recorded at each contrast level. The Kullback-Leibler divergence was estimated according to:
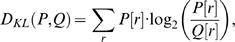
(4)with *r* the response amplitude given as the deviation from the average membrane potential at background light intensity; P and Q are the probability distributions of the response amplitudes for the *reference* and *test responses*, respectively approximated at each data/time point by fitting Gaussians to the response level distributions found across trials. The individual divergences were averaged across time at each contrast level.

### Signal-detection analysis

The discriminability of *reference* and *test responses* was calculated by assessing the response dissimilarity with a simple root-mean-square distance estimation:
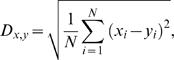
(5)with *x* and *y* being the two responses (either two different *reference responses* or a *reference* and a *test response*), *i* the actual time bin and *N* the length of the data segment, i.e. the number of data points included in the analysis. We chose this distance measure for it is a standard way to collapse the differences between two time-dependent signals into a single value. Basically the same results were obtained when the response similarity was determined on the basis of the absolute value of the difference between *reference* and *test responses*.

Distances were calculated from the same data as used for the Kullback-Leibler divergence (above) according to equation 5. The responses were low-pass filtered with a 500 Hz cut-off which is far beyond the high frequency cut-off of the photoreceptor transfer function. *Reference* and *test responses* were discriminated according to the two average distances 〈*D_r_*〉 and 〈*D_t_*〉. For each single reference response 〈*D_r_*〉 denotes the average distance to all other *reference responses* and 〈*D_t_*〉 the average distance to all *test responses*. In a deterministic system the average distance between an individual *reference response* and all other *reference responses* 〈*D_r_*〉 is zero while the average distance between this *reference response* and the *test responses* 〈*D_t_*〉 is larger than zero. This would be the same for each *reference response* and hence, the reference responses would be assumed discriminable from the test responses with 100% correct performance. In a real system, however, noise originating from different sources corrupts the neuronal response reliability and as a consequence a separation of responses might not be so easy. If the noise in the system is very large compared to the stimulus-induced responses also the *reference responses* appear to be different at each presentation, though evoked by the same stimulus sequence. In this case 〈*D_r_*〉 is not necessarily smaller than 〈*D_t_*〉. In the extreme case, 50% of the *reference responses* can be expected to have a 〈*D_r_*〉 that is smaller than the corresponding 〈*D_t_*〉. The response discriminability then drops to chance level, i.e. 50%. In intermediate cases, with not such a strong noise, the discrimination performance is defined as the percentage of reference responses for which 〈*D_r_*〉 is smaller than 〈*D_t_*〉. When the difference between 〈*D_r_*〉 and 〈*D_t_*〉 was smaller than could be expected from electrical noise (originating form the recording setup) the according response were classified indistinguishable.

The data points for the discrimination performance were fitted with a sigmoid function of the form:
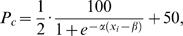
(6)with P_c_ the discrimination performance, x_i_ the contrast, α the slope, and β the position of the infection point. The function is scaled to range from 50 to 100% correct.
